# Network structure-function coupling and neurocognition in cerebral small vessel disease

**DOI:** 10.1016/j.nicl.2023.103421

**Published:** 2023-04-28

**Authors:** Jonathan Tay, Marco Düring, Esther M.C. van Leijsen, Mayra I. Bergkamp, David G. Norris, Frank-Erik de Leeuw, Hugh S. Markus, Anil M. Tuladhar

**Affiliations:** a Stroke Research Group, Department of Clinical Neurosciences, University of Cambridge, Cambridge, UK; b Medical Image Analysis Center (MIAC AG) and qbig, Department of Biomedical Engineering, University of Basel, Basel, Switzerland; c Netherlands Court of Audit, The Hague, the Netherlands; d Department of Neurology, Donders Center for Medical Neurosciences, Radboud University Nijmegen Medical Centre, Nijmegen, the Netherlands; e Centre for Cognitive Neuroimaging, Donders Institute for Brain, Cognition and Behaviour, Radboud University, Nijmegen, the Netherlands

**Keywords:** Small vessel disease, Cognition, MRI, Network analysis

## Abstract

**Background:**

Cerebral small vessel disease is a leading cause of cognitive decline and vascular dementia. Small vessel disease pathology changes structural brain networks, but its impact on functional networks remains poorly understood. Structural and functional networks are closely coupled in healthy individuals, and decoupling is associated with clinical symptoms in other neurological conditions. We tested the hypothesis that structural–functional network coupling is related to neurocognitive outcomes in 262 small vessel disease patients.

**Methods:**

Participants underwent multimodal magnetic resonance imaging and cognitive assessment in 2011 and 2015. Structural connectivity networks were reconstructed using probabilistic diffusion tractography, while functional connectivity networks were estimated from resting-state functional magnetic resonance imaging. Structural and functional networks were then correlated to calculate a measure of structural–functional network coupling for each participant.

**Results:**

Lower whole-brain coupling was associated with reduced processing speed and greater apathy both cross-sectionally and longitudinally. In addition, coupling within the cognitive control network was associated with all cognitive outcomes, suggesting that neurocognitive outcomes in small vessel disease may be related to the functioning of this intrinsic connectivity network.

**Conclusions:**

Our work demonstrates the influence of structural–functional connectivity network decoupling in small vessel disease symptomatology. Cognitive control network function may be investigated in future studies.

## Highlights

• Relationship between structural (SCN) and functional connectivity network (FCN) in small vessel disease (SVD) is unknown.
• Lower SCN-FCN coupling was associated with higher burden of SVD.
• Whole-brain and cognitive control network SCN-FCN coupling was associated with psychomotor speed and apathy.

## 1 Introduction

Cerebral small vessel disease (SVD) is a leading cause of cognitive decline and vascular dementia (Ter Telgte et al., 2018). SVD is associated with altered white matter microstructure that may precede structural connectivity network (SCN) changes in the brain (Lawrence et al., 2014). SCN changes have been associated with clinical symptoms of SVD, including deficits in general cognition, processing speed (PS), and apathy (Tay et al., 2019, Tuladhar et al., 2020). Correlations between SCN changes, SVD-related brain markers, and cognitive symptoms have also been observed in the general population, suggesting that SVD-related brain changes may lead to SCN-mediated cognitive impairment across a range of disease severity (Shen et al., 2020).

Despite these advances, little is known about how SCN changes lead to clinical symptomatology. One explanation lies in the relationship between SCNs and functional connectivity networks (FCNs). FCNs are closely related to cognitive abilities throughout the lifespan and are closely coupled with SCNs (Honey et al., 2009, Sala-Llonch et al., 2015). Accordingly, decreased SCN-FCN coupling has been associated with clinical symptoms in neurological diseases such as Alzheimer's disease (Dai et al., 2019), epilepsy (Zhang et al., 2011), and clinically isolated syndrome (Koubiyr et al., 2019). Furthermore, impaired FCN efficiency has been associated with a greater burden of SVD pathology and impaired cognition (Wang et al., 2022). It is therefore possible that disrupted SCN-FCN coupling may also be associated with cognitive outcomes in SVD, but this has yet to be investigated.

After reconstructing SCNs and FCNs using a multimodal MRI pipeline, we examined how participant-level SCN-FCN coupling correlated with three prominent neurocognitive symptoms of SVD in a prospective cohort: general cognition, PS and apathy (Tay et al., 2020a, Ter Telgte et al., 2018). We also examined SCN-FCN coupling within intrinsic connectivity networks (ICNs) to ascertain network-level effects. Finally, we assessed whether these effects persisted longitudinally using mixed modelling. Based on previous work in other neurological diseases, we hypothesised that lower whole-brain SCN-FCN coupling will be correlated with more severe neurocognitive symptoms. Furthermore, due to the importance of the default mode and central executive networks in human cognition (Sala-Llonch et al., 2015, Seeley et al., 2007), we expected the cognitive effects of SCN-FCN decoupling to be localised to the default mode and cognitive control networks, and that these results would persist longitudinally.

## 2 Material and methods

### 2.1 Participants

The Radboud University Nijmegen Diffusion tensor and Magnetic resonance Cohort (RUN DMC) study is a prospective cohort study on the causes and consequences of SVD (van Norden et al., 2011). Participants were consecutively referred to the Department of Neurology at Radboud University from 2002 to 2006 for acute or subacute symptoms of SVD. Included participants were between 50 and 85 years old and had evidence of SVD on neuroimaging, defined as white matter hyperintensities (WMH) or lacunes of presumed vascular origin (Wardlaw et al., 2013). Baseline data was collected in 2006, with two follow-up assessments in 2011 and 2015. A detailed description of the patient recruitment and study rationale of the study has been described in the study protocol (van Norden et al., 2011). Due to an MRI upgrade between the baseline assessment and first follow-up, only participants with MRI data from 2011 onward were included. The RUN DMC study was approved by the Medical Review Ethics Committee region Arnhem-Nijmegen, and all participants provided written informed consent.

### 2.2 Clinical measures

Clinical measures included assessments of cognitive function and apathy. Cognitive function was assessed using an extensive battery of neuropsychological tests including the Mini Mental State Examination, Rey Auditory Verbal Learning Test, Rey Complex Figure Task, Paper-Pencil Memory Scanning Task (PPMST), an adapted version of the Stroop Color-Word Test, Letter Digit Substitution Task (LDST), a verbal fluency task in which as many animals as possible have to be named within 60 s, followed by as many professions within 60 s, and the Verbal Series Attention Test. Raw scores were converted into z-scores based on the means and standard deviations of the baseline population. These were then averaged to produce a measure of general cognitive function. Z-scores for the PPMST and LDST were also calculated from normative data, then averaged to produce a composite measure of processing speed (PS), which has been shown to be sensitive to SVD in previous studies (Baykara et al., 2016). The cognitive assessment has been fully detailed elsewhere (van Norden et al., 2011). Apathy was assessed using the Apathy Evaluation Scale and depression with the Center for Epidemiologic Studies Depression Scale (Marin et al., 1991, Radloff, 1977). For both measures, higher scores indicate greater apathy or depression.

### 2.3 MR imaging

Imaging data was acquired on a Siemens Magnetom Avanto Tim 1.5 T MRI scanner (Erlangen, Germany). The protocol included a three-dimensional T1-weighted magnetization-prepared rapid gradient-echo (MPRAGE) sequence (repetition time (TR) = 2250 ms, echo time (TE) = 2.95 ms, inversion time (TI) = 850 ms, flip angle = 15°, voxel size = 1.0 mm isotropic), a fluid-attenuated inversion recovery (FLAIR) sequence (TR/TE/TI = 14240/89/2200 ms, voxel size = 1.2 × 1.0 × 2.5 mm, interslice gap = 0.5 mm), a diffusion-weighted imaging (DWI) sequence (TR/TE = 10200/95 ms, voxel size = 2.5 mm isotropic; 7 scans with b = 0 s/mm^2^, 61 scans with b = 900 s/mm^2^), and an eyes-closed resting-state T2*-weighted gradient-echo sequence (TR = 2400 ms, TE = 40 ms, voxel size = 3.5 × 3.5 × 4.0 mm, flip angle = 90°, fat saturated, interleaved ascending acquisition).

### 2.4 SVD markers

To assess relationships between SCN-FCN coupling and SVD pathology, we measured four markers of SVD: lacunes, WMH, microbleeds, and peak width of skeletonised mean diffusivity (PSMD) (Baykara et al., 2016, Wardlaw et al., 2013). The number of lacunes and microbleeds were manually rated on FLAIR/T1-weighted and T2*-weighted MRI scans according to the STRIVE criteria (Wardlaw et al., 2013) by 2 trained raters blind to the clinical data. Follow-up FLAIR images were resliced to match the baseline scans to prevent differences in partial volume effects between baseline and follow-up scans. Inter-rater and intra-rater reliability were good, with a weighted Cohen’s *κ* of 0.87 and 0.95 for lacunes and 0.85 and 0.96 for microbleeds, respectively. WMH were segmented on FLAIR images with a semiautomatic method and PSMD was derived from DWI images, as described previously (Baykara et al., 2016, van Norden et al., 2011).

### 2.5 Network construction

**Fig. 1:**
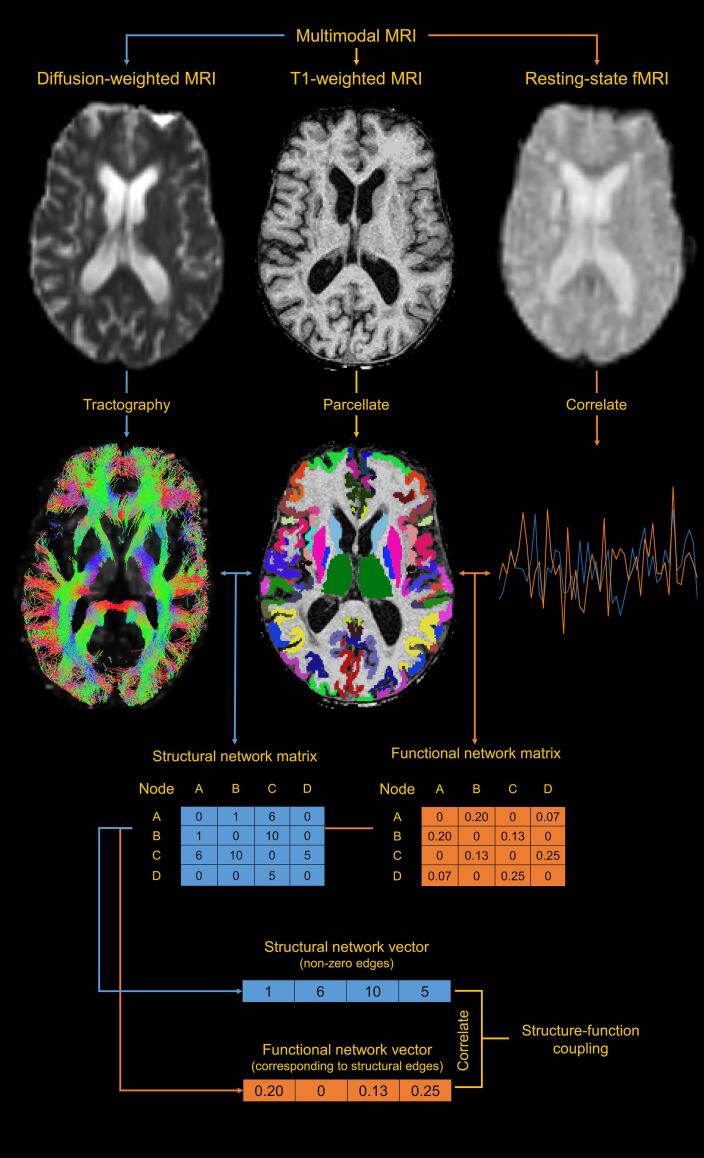
The network analysis pipeline. Multimodal MRI is used to acquire T1-weighted images, diffusion weighted images (DWI), and resting-state functional MRI (fMRI) sequences. The T1-weighted image is anatomically parcellated to obtain network nodes. Probabilistic tractography is used on DWI data to derive structural network edges. Resting-state fMRI signals are correlated between regions to derive functional network edges. The node and edge images are then combined to form individual symmetric structural and functional connectivity matrices. Non-zero edges are extracted from the structural connectivity matrix, along with corresponding edges in the functional connectivity matrix. The correlation between the resulting structural and functional connectivity vectors is a measure of structure–function coupling.

A network is composed of two elements: nodes and connecting edges. In the context of MRI-based network analysis, nodes are defined as anatomical regions, while edges are the structural or functional connections between those regions (Bullmore and Sporns, 2009). In our study, structural networks were reconstructed using DWI data, while functional networks were estimated using resting-state fMRI (Fig. 1). This required pre-processing for T1w, DWI, and fMRI data.

### 2.6 T1w pipeline and node definition

All participant T1w images at all available timepoints were processed using FreeSurfer 6.0 (surfer.nmr.mgh.harvard.edu). FreeSurfer is an automated software suite for processing T1w images that includes: skull stripping, registration to standard space, segmentation, surface reconstruction, and cortical parcellation. In the longitudinal stream, these are performed on robustly-registered within-participant template images, improving registration and parcellation accuracy (Reuter et al., 2012). Atlas nodes were defined using the Destrieux parcellation (Destrieux et al., 2010), combined with subcortical nodes from automated segmentation. This yielded 162 regions (81 per hemisphere) to use as network nodes for further analysis.

### 2.7 DWI pipeline

The pipeline for processing raw diffusion data included: (1) denoising using a local principal component analysis filter (Manjon et al., 2013), (2) correction for head movement, cardiac motion and eddy currents using the PATCH algorithm (Zwiers, 2010), (3) unwarping of susceptibility distortions by normalizing the images to the T1w images in the phase-encoding direction using SPM12 (fil.ion.ucl.ac.uk/spm/software/spm12), (4) brain tissue extraction using BET (Smith, 2002), and (5) co-registration to the T1w image using boundary-based registration (BBR) (Greve and Fischl, 2009).

Tractography was conducted using MRtrix 3.0 (mrtrix.org). White matter response functions were estimated from the processed DWI images and used to calculate voxelwise fibre orientation distribution (FOD) functions using single-shell single-tissue constrained spherical deconvolution (Tournier et al., 2007, Tournier et al., 2013). The resulting FOD images were used for anatomically constrained tractography (ACT). The ACT framework uses anatomical priors to increase the biological accuracy of reconstructed streamlines while reducing false positives (Smith et al., 2012). ACT requires masks of the cortical GM, subcortical GM, WM, and CSF, and lesioned tissue. The first four masks were obtained from each participant's longitudinal FreeSurfer segmentation, while the mask of lesioned tissue was defined as WMH identified on FLAIR sequences (Tuladhar et al., 2020).

Probabilistic streamlines were seeded at the WM-GM boundary and computed using the second-order integration over fibre orientation distributions algorithm. Termination criteria included: minimum length < 10 mm, maximum length > 250 mm, turning angle > 45°, FOD amplitude < 0.1, or entering the CSF (Smith et al., 2012). Backtracking was enabled to re-compute streamlines if poor structural terminations were encountered. Seeding proceeded until 10 million valid streamlines were generated. These were then filtered to 1 million streamlines using the spherical-deconvolution informed filtering of tractograms (SIFT) algorithm to reduce reconstruction bias and further improve biological accuracy (Smith et al., 2013).

### 2.8 Structural network construction

Filtered tractograms from SIFT were used to estimate structural network connectivity in each participant. Edges were defined as the number of streamlines with endpoints in two different nodes. This yielded a 162 × 162 structural connectivity matrix for each participant, where each node was an anatomical region and each edge was the number of reconstructed streamlines connecting them. These matrices were then masked to include edges that appeared in at least 90% of the population. Structural edge weights were then log(x + 1) transformed to normalize positively skewed distributions.

### 2.9 Resting-state pipeline

The pipeline for processing resting-state data included: (1) removal of the first four volumes of each acquisition to allow for steady-state magnetization, (2) motion parameter estimation and realignment using MCFLIRT (Jenkinson et al., 2002), (3) brain tissue extraction using BET, (4) co-registration to the T1w image using BBR, (5) demeaning and linear detrending, (6) temporal filtering using a Butterworth filter with a passband between 0.009 and 0.08 Hz, and (7) 36-parameter regression using 9 base regressors (the 6 rigid-body motion parameters estimated with MCFLIRT, mean WM signal, mean CSF signal, and global signal), their first-order temporal derivatives, quadratic terms, and the squares of their derivatives. Steps (6) and (7) were applied orthogonally to avoid reintroducing artifacts that can arise from serial application (Lindquist et al., 2019). Global signal regression (GSR) is a controversial pre-processing step in fMRI pipelines, but was included in (7) due to its robust correction for motion, cardiac, and respiratory signals (Murphy and Fox, 2017), which were a concern given our older population.

This approach has been adapted from pipelines that are effective at removing motion-related artifact in functional connectivity studies (Ciric et al., 2017, Lydon-Staley et al., 2019). Furthermore, the steps used in this pipeline have been shown to mitigate low-frequency BOLD signal fluctuations driven by upstream changes in cerebral haemodynamics (Zhu et al., 2015). All processing was done using FSL 6.0.1 and functions in Python 3.7.5 with Nilearn 0.5.2.

### 2.10 Functional network construction

For each participant, mean BOLD signal intensities were extracted per-region across the processed time series. Pearson correlation coefficients were then computed for all pairs of regions. Fisher's Z transformation was applied to all coefficients to further normalize values. Given the controversial interpretation of anticorrelated resting-state signals, negative correlations were set to 0 (Dai et al., 2019, Koubiyr et al., 2019). This yielded a 162 × 162 functional connectivity matrix for each participant, where each node was an anatomical region and each edge was a positive Fisher-normalized Pearson correlation between the BOLD signal intensities for those regions.

### 2.11 Community structure

Communities are groups of nodes that are densely intraconnected but minimally interconnected. In the context of brain networks, communities reflect latent organizational principles of the brain such as intrinsic connectivity (Rubinov and Sporns, 2010). Examining SCN-FCN coupling within these communities may reveal which networks are related to cognitive outcomes in SVD.

Given that community membership differs between participants, we examined community structures at the group level by averaging the FCNs of all participants in 2011. This group-average FCN was proportionally thresholded to retain 10% edge density. Functional communities were detected in the group-average FCN using the Louvain algorithm, which partitions nodes into communities by maximizing a metric known as the modularity (Blondel et al., 2008, Newman, 2006). This algorithm is non-deterministic, so we repeated this modular decomposition 1000 times, using the results to construct an agreement matrix where elements corresponded to the probability of a pair of nodes being in the same community. This agreement matrix was used to generate a consensus partition, which is a stable community structure of the group network (Lancichinetti and Fortunato, 2012). The final modularity of the consensus partition was 0.64. Community detection and consensus partitioning were carried out using functions implemented in the Brain Connectivity Toolbox (BCT) in MATLAB R2019a (Mathworks) (Rubinov and Sporns, 2010).

### 2.12 Structure-function coupling

The SCN-FCN coupling coefficient was defined as the Pearson correlation between non-zero edges of an individual's SCN and their corresponding FCN. We restricted our analysis to edges that appeared in 90% of all SCNs for participants in 2011. This resulted in a single number representing the strength of the relationship between structural and functional network topology. SCN-FCN coupling was also calculated for the communities delineated earlier.

### 2.13 Global efficiency

Structural network efficiency may mediate associations between SVD pathology and outcomes (Tay et al., 2019, Tuladhar et al., 2020). To control for the effects of this on SCN-FCN coupling, we calculated the global efficiency of structural networks using the BCT for use as a covariate in subsequent analyses. Global efficiency is the average inverse shortest path length of a network, and measures integration within the brain (Rubinov and Sporns, 2010).

### 2.14 Statistical analysis

Statistics were calculated using functions and packages in R 4.0.3. Analyses were two-tailed with *ɑ* = 0.05, and multiple comparisons were corrected using the false discovery rate (FDR).

Data was missing for some clinical variables. In 2011, data was missing for cognition (n = 2), PS (n = 5), apathy (n = 10), and depression (n = 4). In 2015, data was missing for PS (n = 2), apathy (n = 1), and depression (n = 5). Missing values were imputed using the mice package 3.13.0. The imputed dataset included all demographic variables, clinical measures, and MRI measures. Predictive mean matching was used to generate 20 imputations with a random seed offset of 1234. For linear regression and mixed effect models, model estimates for each imputation were averaged and the total variance over repeated analysis pooled using Rubin’s rules.

Linear regression models were used to test cross-sectional associations between SCN-FCN coupling and several dependent variables. First, we assessed associations with MRI markers of SVD: lacunes, WMH, microbleeds, and PSMD. Next, we tested associations with cognitive outcomes: general cognition, PS, and apathy. Depression was also tested to assess the specificity of SCN-FCN coupling to cognitive outcomes, as previous work shows weak associations between depression and cognition in SVD (Tay et al., 2020b). To ensure these results were not dependent on demographic factors or structural network efficiency, age, sex, and global efficiency were added as covariates to these models.

We then used linear mixed-effect models to assess longitudinal relationships between SCN-FCN coupling and the abovementioned outcomes using the lme4 package 1.1–26. Mixed-effect models assess population-level and individual-level effects using fixed and random effects, respectively. Models assessed group-level longitudinal correlations between SCN-FCN coupling and outcomes while controlling for time between follow-up, age, sex, and global efficiency. A participant-level random intercept was added to control for individual variance in initial values.

### 2.15 Data availability

Anonymized data will be made available upon reasonable request to the corresponding author.

## 3 Results

### 3.1 Participant characteristics

**Fig. 2:**
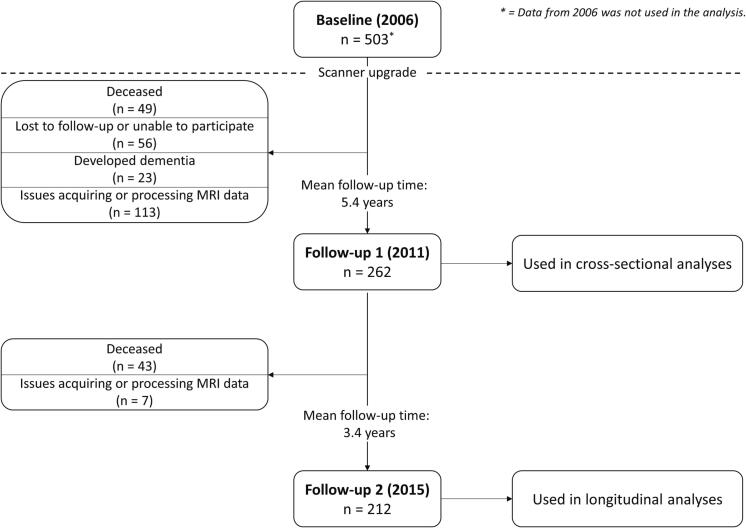
Participant flow in RUN DMC. Due to a scanner upgrade after 2006, only participant data from 2011 onwards is used.

At baseline in 2006, 503 participants were enrolled into RUN DMC. Of these, 262 had usable imaging data at the 2011 follow-up, while 212 had usable data in 2015 (Fig. 2). Descriptive statistics were calculated for participants at both timepoints (Table 1).

**Table 1:** Descriptive statistics for participants in 2011 and 2015.

	Cross-sectional (n = 262)	Longitudinal (n = 212)
Age, mean (SD)	67.8 (8.0)	70.0 (7.5)
Sex, Female, n (%)	114 (43.5%)	91 (42.9%)
Education, median (IQR)	10.0 (5.0)	10.0 (5.0)
Hypertension, n (%)	207 (79.0%)	183 (86.3%)
Diabetes, n (%)	31 (11.8%)	25 (11.8%)
Hypercholesterolemia, n (%)	112 (42.7%)	95 (44.8%)
Smoking, n (%)		
Never	81 (30.9%)	65 (30.7%)
Former	148 (56.5%)	120 (56.6%)
Current	33 (12.6%)	27 (12.7%)
BMI, mm/kg^2^, mean (SD)	27.9 (4.8)	27.1 (4.0)
WMH, cm^3^, mean (SD)	6736.6 (9942.2)	8289.4 (11336.4)
General cognition, mean (SD)	0.2 (0.7)	0.3 (0.7)
PS, mean (SD)	−1.1 (0.7)	−0.9 (0.7)
Apathy (log), mean (SD)	3.2 (0.2)	3.0 (0.2)
Depression (log), mean (SD)	2.7 (0.3)	2.7 (0.2)
Structure-function coupling, mean (SD)		
Whole-brain network	0.22 (0.04)	0.22 (0.04)
Salience network	0.19 (0.10)	0.21 (0.11)
Memory network	0.22 (0.09)	0.19 (0.09)
Visual network	0.17 (0.10)	0.28 (0.08)
Cognitive control network	0.24 (0.07)	0.24 (0.06)
Default mode network	0.14 (0.10)	0.12 (0.10)

*Note*. BMI = body mass index; WMH = white matter hyperintensities; PS = processing speed.

### 3.2 Structural and functional networks in SVD

**Fig. 3:**
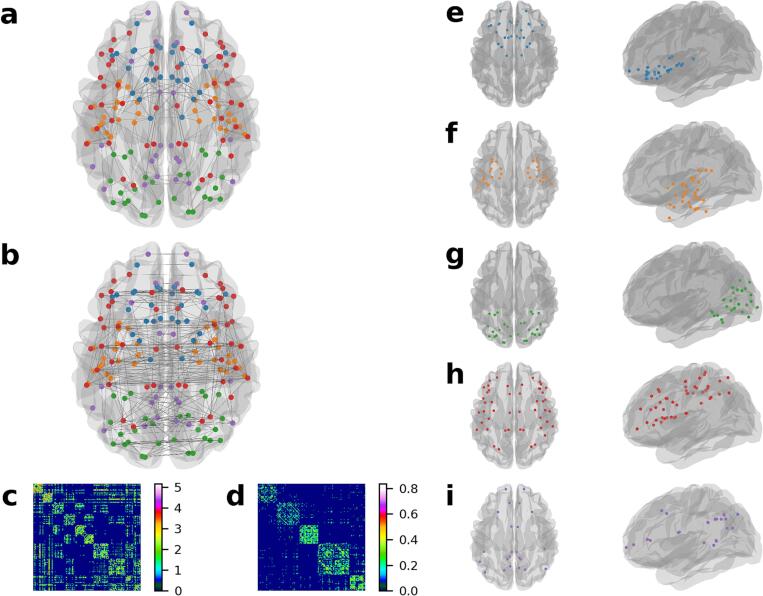
Structural and functional networks in cerebral small vessel disease. a-b, Group average networks displaying top 2.5% of connections, coloured by community membership. a, Structural network derived using probabilistic diffusion tractography. This network is characterised by several short connections with a few long-range connections. b, Functional network derived using resting-state functional MRI. The strongest connections in this network are bilateral interhemispheric connections. c-d, Group average network matrices ordered by community membership. The order appears in Supplementary Tables 1–5. c, Group average structural network, with edge weights log transformed. d, Group average functional network with 10% connection density. e-i, Consensus partition of functional communities delineated using the Louvain algorithm, with a final modularity of 0.64. e, Salience network. f, Memory network. g, Visual network. h, Frontoparietal cognitive control network. i, Default mode network.

The group-averaged structural network was characterised by short and predominantly intra-hemispheric connections (Fig. 3a). In contrast, all nodes in the average functional network exhibited low to moderate correlations, with a few very strong inter-hemispheric connections (Fig. 3b). These are reinforced by their respective connectivity matrices (Fig. 3c,d).

Modular decomposition of functional networks revealed five distinct communities (Fig. 3e-i; Supplementary Tables 1–5). These were classified, based on previous literature, as the salience network (Fig. 3e), memory network (Fig. 3f), visual network (Fig. 3g), frontoparietal cognitive control network (Fig. 3h), and default mode network (DMN; Fig. 3i) (Uddin et al., 2019). Coupling within these communities showed moderate to strong correlations with whole-brain coupling (r = 0.38–0.64), as well as weak to moderate correlations with other communities (Table 2).

**Table 2:** Modular structure–function coupling correlations.

	Whole-brain	Salience	Memory	Visual	Executive	DMN
Whole-brain		**0.52**	**0.38**	**0.55**	**0.64**	**0.48**
Salience			0.04	0.11	**0.21**	0.12
Memory				**0.14**	**0.30**	**0.17**
Visual					**0.30**	**0.28**
Control						**0.17**
DMN						

*Note*. Bold numbers refer to correlations significant at *P_FDR_* < 0.05. DMN = default mode network.

### 3.3 Structure-function coupling and SVD burden

After controlling for age, sex, and structural network efficiency, and correcting for the FDR, models revealed that whole-brain network SCN-FCN coupling was not associated with SVD markers cross-sectionally or longitudinally (Table 3, Table 4). Subnetwork-specific analysis revealed SCN-FCN coupling in the cognitive control network was associated with lower PSMD, which was consistent in longitudinal models, although not after correction for multiple comparisons (Table 4). Unadjusted results are in Supplementary Tables 6–7.

**Table 3:** Linear regression estimates between structure–function coupling in subnetworks, SVD markers and neurocognitive symptoms after controlling for age, sex, and global efficiency.

	Lacunes	WMH	Microbleeds	PSMD (10^−4^)	Cognition	PS	Apathy	Depression
Whole	−0.26	−26155.56	−0.77	−1.49	1.55*	2.2^**^	−0.83^**^	−1.73
Salience	0.14	4494.05	0.15	0.29	0.13	0.05	−0.17	−0.33
Memory	0.07	−7034.94	0.13	−0.57	0.57	0.82*	−0.26*	−1.48*
Visual	0.1	−1370.27	0.18	0.22	−0.04	0.15	−0.09	0.33
Control	−0.21	6754.75	−0.19	−1.17^**^	1.75^**^	2.37^**^	−0.57^**^	−1.39
DMN	−0.04	−5006.69	−0.53*	0.39	−0.06	−0.13	0.07	0.03

*Note*. Numbers are unstandardised beta coefficients. * = significant at *P_uncorrected_* < 0.05, ** = significant at *P_FDR_* < 0.05. Whole = whole-brain structure–function coupling, DMN = default mode network, PS = processing speed, WMH = white matter hyperintensities normalised for head size; PSMD = peak width of skeletonised mean diffusivity. Apathy, depression, lacunes, and microbleeds are natural log transformed.

**Table 4:** Linear mixed-effect estimates between structure–function coupling in subnetworks, SVD markers and neurocognitive symptoms after controlling for age, sex, and global efficiency, and follow-up time.

	Lacunes	WMH	Microbleeds	PSMD (10^−4^)	Cognition	PS	Apathy	Depression
Whole	−0.45	−29052.38	−0.47	−1.8*	0.91	2.28^**^	−1.02^**^	−0.45
Salience	−0.04	8601.94	0.22	0.4	−0.06	0.17	−0.2	−0.26
Memory	0.09	−7761.81	0.06	−0.5	0.17	0.26	−0.05	−0.43
Visual	0	3168.87	0.04	−0.01	0.49^**^	0.85^**^	−0.67^**^	−0.95^**^
Control	−0.03	−4448.97	−0.21	−0.99*	0.87^**^	1.28^**^	−0.47^**^	−0.26
DMN	0.1	−2983.32	−0.32	0.17	−0.04	0.11	0.03	0.15

*Note*. Numbers are unstandardised beta coefficients. * = significant at *P_uncorrected_* < 0.05, ** = significant at *P_FDR_* < 0.05. Whole = whole-brain structure–function coupling, DMN = default mode network, PS = processing speed, WMH = white matter hyperintensities normalised for head size; PSMD = peak width of skeletonised mean diffusivity. Apathy, depression, lacunes, and microbleeds are natural log transformed.

### 3.4 Structure-function coupling and neurocognitive outcomes

Cross-sectional models revealed that whole-brain network SCN-FCN coupling was associated with higher PS and lower apathy, while control network coupling was associated with cognition, PS, and negatively associated with apathy (Table 3). The memory network was associated with PS, apathy, and depression, although not after correction for multiple comparisons. Findings for whole-brain and control network SCN-FCN coupling were consistent in longitudinal models (Table 4). Longitudinally, the memory network was not associated with any outcome, and visual network coupling became associated with cognition, PS, apathy, and depression.

## 4 Discussion

The main finding of this study is that SCN-FCN coupling is associated with microstructural disease burden and neurocognitive outcomes in SVD patients both cross-sectionally and longitudinally. Specifically, SCN-FCN coupling within the frontoparietal cognitive control network, after controlling for demographic factors and structural network efficiency, was associated with lower PSMD, a marker sensitive to SVD progression (Baykara et al., 2016), despite not being associated with visible markers of SVD such as lacunes or WMH. This suggests that SCN-FCN coupling is more strongly associated with microstructural damage in SVD, which may be more indicative of cognitive deficits than traditional radiological markers of SVD (Lawrence et al., 2014). Additionally, whole-brain and control network coupling was cross-sectionally and longitudinally associated with PS and apathy, two major symptoms of SVD, suggesting that neurocognitive outcomes may be related to the functioning of these networks. By controlling for global efficiency, we show that these results explain variance in outcomes beyond SCNs alone.

We found that SCN-FCN coupling within the cognitive control network was consistently associated with neurocognitive outcomes, supporting one of our hypotheses. Other ICNs were not consistently associated with outcomes, suggesting that the associations between whole-brain coupling and outcomes are primarily driven by coupling within the control network. Previous research has demonstrated control network activity during cognitively demanding tasks in healthy individuals (Seeley et al., 2007). Lower SCN-FCN coupling within this network, which may be driven by age and disease-related factors, may manifest as SVD-related neurocognitive deficits. Our longitudinal results suggest that this process continues through time, with further decoupling leading to greater cognitive impairment. Importantly, the control network was not associated with depression, supporting findings that depression is not strongly related to cognitive outcomes in SVD (Tay et al., 2020b).

In contrast, DMN coupling was not correlated with any outcome, which was unexpected given reported associations between suppressed functional activity within DMN and cognitive deficits in other diseases (Anticevic et al., 2012). This may be due to the ability of the DMN to rapidly reconfigure its topology following brain injuries such as stroke (Park et al., 2014), which can occur faster than corresponding changes to white matter organisation. Indeed, functional compensation has been observed as early as 3 months post-stroke, a period during which secondary white matter neurodegeneration is still occurring (Grefkes and Ward, 2014).

Our differential network results may be a potential biomarker for SVD and suggest possible avenues for treatment. For instance, some have suggested that the function of specific brain networks can be modulated by techniques such as transcranial magnetic stimulation (Fox et al., 2012). This technique could potentially be applied to improve control network activity in SVD patients, potentially increasing SCN-FCN coupling within these networks and ameliorating cognitive impairment and apathy (Tay et al., 2020a).

One explanation for the difference in findings regarding DMN and the control network may be what decreasing SCN-FCN coupling represents for both ICNs. Within DMN, lower correlations between structural and functional networks may represent functional reorganisation along indirect white matter pathways. Reorganisation here may reflect compensation in other domains that do not rely as heavily on cognition, such as functional abilities, which are also impaired in SVD (Ter Telgte et al., 2018). Other work found that functional hub nodes, which are highly connected with other nodes, differed in SVD patients compared to healthy controls, which is further evidence of partial reorganisation to rebalance brain function in SVD (Xin et al., 2022). Decreasing coupling within the control network, however, may represent reductions in either structural or functional connectivity, which are not compensated for, resulting in impaired neurocognition.

Another explanation for SCN-FCN decoupling may lie in SVD-related changes to the BOLD response. Preliminary work has shown that graph-based metrics of functional connectivity in SVD patients have limited test–retest reliability (Gesierich et al., 2020, Lawrence et al., 2018). The reasons for this are debated, with some suggesting that SVD changes the BOLD response by impairing neurovascular coupling (Lawrence et al., 2018), while others suggest that age-related pathologies play a more important role than SVD (Gesierich et al., 2020). We attempted to compensate for both of these explanations in several ways, including: a robust fMRI pre-processing pipeline, only considering FCN edges with corresponding SCN edges, as SCNs show high reproducibility (Lawrence et al., 2018), and controlling for age and global efficiency in our statistical analyses, as these are sensitive to SVD pathology (Lawrence et al., 2014). Importantly, the community structure of resting-state networks within our population was consistent with those found in healthy individuals (Sala-Llonch et al., 2015, Seeley et al., 2007), suggesting that impaired neurovascular coupling may not substantially alter the fundamental topology of FCNs. Finally, the associations between SCN-FCN coupling and cognition suggests that the measure has some biological relevance despite the possible limitations.

One important caveat to interpreting our results relates to the functional communities we identified. Although these are broadly consistent with macro-scale functional networks reported in the literature (Uddin et al., 2019), our study does diverge somewhat in the number of communities detected, as well as their overall topology. Some of these differences may be related to the brain parcellation we used to derive network matrices, which delineates brain regions based on gyral and sulcal morphology (Destrieux et al., 2010). This approach yields nodes that are interpretable, but at the cost of more detailed resolution. In contrast, other studies that have used smaller regions – for example, by uniform sampling the cortical surface – have shown that a greater number of networks can be delineated (Yeo et al., 2011). It is possible that SCN-FCN coupling within these fine-grained networks could show stronger correlations with SVD pathology and cognition when compared to our large-scale networks, which future studies could investigate in more detail. That said, the number of networks we detected is close to commonly accepted macro-scale functional networks (Uddin et al., 2019), and consistent with other clinical studies that use the same atlas (Koubiyr et al., 2019).

Another factor that may have influenced the communities detected, as well as the interpretation of our results, is our use of GSR. GSR is controversial pre-processing technique because it artificially shifts functional correlations towards negative values, potentially altering statistical relationships between nodes (Murphy and Fox, 2017). This may have been the case in our study, and could have led to changes in functional network dynamics that might have subsequently influenced community detection, which could explain differences in results compared to other studies. Despite this, we deemed GSR an important step for its robust correction of motion artifacts, which was a concern in our sample, which was older and had SVD pathology, which may lead to motor impairment (Su et al., 2018). Furthermore, the networks delineated in our sample were broadly consistent with others reported in the literature (Uddin et al., 2019), including other studies examining resting-state functional networks in SVD (Xin et al., 2022). We have chosen to not repeat analyses without GSR given that a systematic review of functional network studies in SVD found that the technique had a minimal impact on specific patterns of functional connectivity or subsequent correlations with cognition (Schulz et al., 2021). Furthermore, examining results using different pre-processing steps was outside the scope of the current study, and may be better suited for future work focused on examining the effect of different fMRI pre-processing pipelines on functional network connectivity in SVD.

Our study had other limitations. We only had two timepoints to use in longitudinal analyses, which allowed us to quantify the direction of coupling but not its rate of change, which requires more timepoints. Rates of change may be relevant given nonlinear increases in SVD pathology over time (van Leijsen et al., 2019). Second, we only considered functional connections with underlying structural connections. Areas without structural connections can be functionally connected through indirect paths (Honey et al., 2009), and more advanced computational methods are needed to assess the effects of these on SCN-FCN coupling. Finally, our scans were acquired on a 1.5 T MRI, a relatively low field strength.

In conclusion, we showed that decreases in SCN-FCN coupling are associated with a range of neurocognitive outcomes in SVD. In particular, decreasing SCN-FCN coupling within the cognitive control network was associated with deficits in general cognition, PS, and greater apathy, in cross-sectional and longitudinal analyses. Future network studies on the pathophysiology and treatment of SVD could focus on this specific ICN, which may have important implications for outcome.

## CRediT authorship contribution statement

**Jonathan Tay:** Conceptualization, Methodology, Formal analysis, Writing – original draft. **Marco Düring:** Writing – review & editing. **Esther M.C. van Leijsen:** Writing – review & editing, Data curation, Investigation. **Mayra I. Bergkamp:** Writing – review & editing, Data curation, Investigation. **David G. Norris:** Writing – review & editing. **Frank-Erik de Leeuw:** Writing – review & editing, Project administration. **Hugh S. Markus:** Writing – review & editing. **Anil M. Tuladhar:** Writing – review & editing, Supervision.

## Declaration of Competing Interest

The authors declare that they have no known competing financial interests or personal relationships that could have appeared to influence the work reported in this paper.

## Data Availability

Data will be made available on request.
